# Synthetic Methods, Chemistry, and the Anticonvulsant Activity of Thiadiazoles

**DOI:** 10.1155/2013/348948

**Published:** 2013-04-30

**Authors:** Bhawna Sharma, Amita Verma, Sunil Prajapati, Upendra Kumar Sharma

**Affiliations:** ^1^Institute of Pharmacy, Bundelkhand University, Jhansi 284128, India; ^2^Department of Pharmaceutical Sciences, Faculty of Health Sciences, Sam Higginbottom Institute of Agriculture, Technology and Sciences-Deemed University, Allahabad 211007, India

## Abstract

The chemistry of heterocyclic compounds has been an interesting field of study for a long time. Heterocyclic nucleus 1,3,4-thiadiazole constitutes an important class of compounds for new drug development. The synthesis of novel thiadiazole derivatives and investigation of their chemical and biological behavior have gained more importance in recent decades. The search for antiepileptic compounds with more selective activity and lower toxicity continues to be an active area of intensive investigation in medicinal chemistry. During the recent years, there has been intense investigation of different classes of thiadiazole compounds, many of which possess extensive pharmacological activities, namely, antimicrobial activity, anticonvulsant, antifungal antidiabetic, anti-inflammatory, antioxidant, and antituberculosis activities, and so forth. The resistance towards available drugs is rapidly becoming a major worldwide problem. The need to design new compounds to deal with this resistance has become one of the most important areas of research today. Thiadiazole is a versatile moiety that exhibits a wide variety of biological activities. Thiadiazole moiety acts as “hydrogen binding domain” and “two-electron donor system.” It also acts as a constrained pharmacophore. On the basis of the reported literature, we study here thiadiazole compounds and their synthetic methods chemistry and anticonvulsant activity.

## 1. Introduction

Epilepsy is the name of a brain disorder characterized predominantly by recurrent and unpredictable interruptions of normal brain function, called epileptic seizures [1, 2]. The current therapy of epilepsy with antiepileptic drugs is associated with side effects, dose-related and chronic toxicity, and teratogenic effects [3, 4]. Epilepsy is not a singular disease entity but a variety of disorders reflecting underlying brain dysfunction that may result from many different causes. Therefore, there is continuing demand for new anticonvulsant agents. So, there is an urgent requirement for the dieovery and development of some novel anticonvulsant agents with more selective activity and lower toxicity for the effective treatment of epilepsy. Several five-membered aromatic systems having three heteroatoms at symmetrical positions such as thiadiazoles have been studied extensively owing to their interesting pharmacological activities. There is a broad variety of heterocyclic compounds which are having medicinal importance, and recently, much attention has been focused on thiadiazole derivatives in view of their broad spectrum activities. Thiadiazole is one such heterocyclic nucleus. There are several isomers of thiadiazole, that is 1,2,3-thiadiazole, 1,2,4-thiadiazole, 1,2,5-thiadiazole, and 1,3,4-thiadiazole [5], 1,3,4-Thiadiazole is the main isomer of thiadiazole series having versatile pharmacological activities.

## 2. Thiadiazole

Thiadiazole is a heterocyclic organic compound that has a five-member ring having one sulphur and two nitrogen atoms [6]. 1,3,4-Thiadiazoles represent one of the most biologically active classes of compounds, possessing a wide spectrum of activities. Thiadiazoles have become very important compounds in medicine, agriculture, and many fields of technology. A large number of 1,3,4-thiadiazoles have been patented in the agricultural field as herbicides and bactericides [7].

X-ray analysis shows the following structure parameter for 1,3,4-thiadiazole ring (see Table 1 and Structure **(1)**):


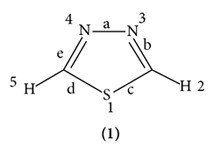


## 3. Chemistry of Thiadiazole

A recent literature survey revealed that the 1,3,4-thiadiazole moiety has been widely used by the medicinal chemist in the past to explore its biological activities. The development of 1,3,4-thiadiazole chemistry is linked to the discovery of phenylhydrazines and hydrazine in the late nineteenth century. The first 1,3,4-thiadiazole was described by Fischer in 1882, but the true nature of the ring system was demonstrated first in 1890 by Freund and Kuh.

Thiadiazole is a five-member heterocyclic compound having one sulphur and two nitrogen atoms. There are several isomers of thiadiazole, that is, 1,2,3-thiadiazole **(2)**, 1,2,5-thiadiazole **(4)**, 1,2,4-thiadiazole **(3)**, and 1,3,4-thiadiazole **(5)**. There are four possible systems in thiadiazole.


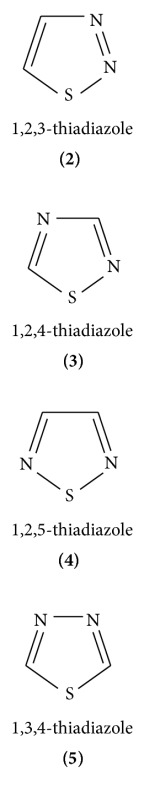


## 4. 1,3,4-Thiadiazoles

1,3,4-Thiadiazole was first described in 1882 by Fischer and further developed by Bush and his coworkers, but true nature of the ring system was demonstrated first in 1956 by Goerdler et al. [8]. The advent of sulphur drugs and the later discovery of mesoionic compound greatly accelerated the rate of progress in the field of thiadiazole. Thiadiazole carrying mercapto, hydroxyl, and amino substituents can exist in many tautomeric forms. The 1,3,4-thiadiazoles are conveniently divided into three subclasses:

- aromatic systems which include the neutral thiadiazoles and constitute a major part of this paper;
- mesoionic systems which are defined as five-membered heterocycles which are not covalent or polar and possess a sextet of electrons in association with the five atoms comprising the ring;
- nonaromatic systems such as the 1,3,4-thiadiazoles and the tetrahydro 1,3,4-thiadiazoles. In the partially reduced systems, the position of the double bond is denoted by the prefix Δ, with being a Δ^2^-1,3,4-thiadiazole (Structures **(6)**, **(7)**, and **(8)**).

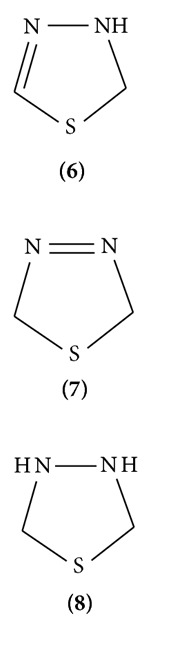


## 5. Synthetic Procedures of 1,3,4-Thiadiazoles

**Scheme 1:**
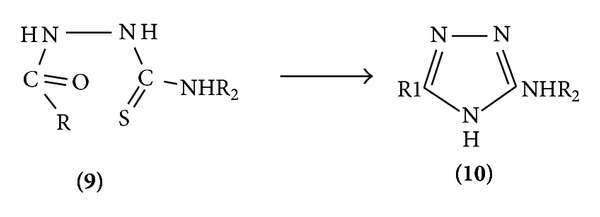


*(a) Formation of One Bond*. The most common procedure for the synthesis of 5-substituted 2-amino-thiadiazole is the acylation of a thiosemicarbazide followed by dehydration. Sulphuric acid, polyphosphoric acid, and phosphorous halides are some of the reagents used. The most recent procedure utilizes 1.5 moles of methane sulphonic as a dehydrating agent and the thiadiazoleare obtained in the high yield and good purity. 5-Alkyl-2-methyl amino-1,3,4-thiadiazoles are prepared from a suitable carboxylic acid and methyl thio-semicarbazide in the presence of three parts of polyphosphoric acid and one part of concentreted sulphuric acid. 2-alkylamino-1,3,4-thiadiazole *n* substituted in the 5th position can be prepared in high yields by the reaction of 4-alkylthiosemicarbazides with orthoformate esters in the presence of small amount of concentreted hydrochloric acid [9] (Scheme 1).

*(b) Formation of Two Bonds*. This is the most widely used procedure for the synthesis of thiadiazoles, thiazolidines, and mesoionic thiadiazoles.

**Scheme 2:**
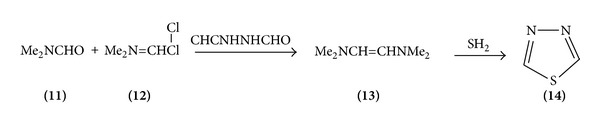


*(1) Cyclization*. The parent molecule 1,3,4-thiadiazole was synthesized in 1956 by a four-step reaction sequence start utilizing hydrazine and from thiosemicarbazide. A second procedure utilizes hydrazine and potassium dithioformate. Dehydration of DMF with thionyl chloride or phosgene gives the formamide chloride which on treatment with N, N′Diformylhydrazine gives the dihydrochloride of the free base which is liberated with sodium ethoxide, which then cyclizes to thiadiazole in the presence of hydrogen sulphide in an overall yield of 65%. 2-Amino 1,3,4-thiadiazole is also prepared from thiosemicarbazide and a mixture of formic and hydrochloric acid in a tedious procedure with an overall yield of 65% (Scheme 2).

*(2) Dipolar Cycloadditions*. This procedure has been widely used during the last decade for both synthetic and mechanistic reasons.

**Scheme 3:**
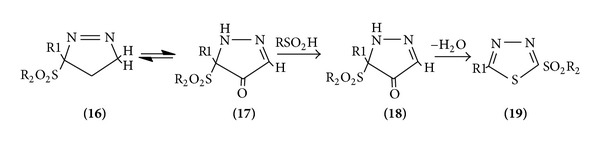


*From Diazo Compounds*. The reaction of aryl sulphonyl-substituted sulfines with diazomethane gives Δ^3^-1,3,4-thiadiazole 1-oxide which, however, is unstable and rearranged via an isomerization of the Δ^3^ to the Δ^2^-thiadiazole oxide. This is followed by an elimination and readdition of sulfonic acid and loss of water in a pummerer-type aromatization to give the rearranged thiadiazole (Scheme 3).

### 5.1. Method of Synthesis of 1,3,4-Thiadiazole

**Scheme 4:**
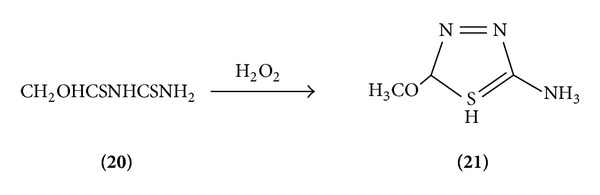


**Scheme 5:**
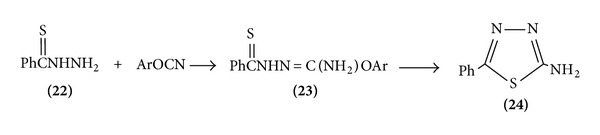


**Scheme 6:**
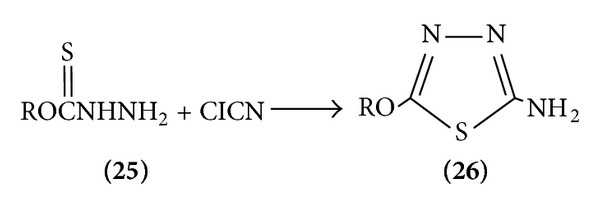


*(a) From Hydrazine*. 3-Thiocarbamoyl thione methyl carbonate on oxidation with H_2_O_2_ gave alkoxythiadiazole [10] (Scheme 4).

(I) Thiobenzoyl hydrazine cyclized to produce 1,3,4-thiadiazole-thiobenzoyl hydrazine [11] (Scheme 5).

(II) Thione carbazate are cyclized by cyanogens chloride or bromide to give 1,3-thiadiazole [12] (Scheme 6).

**Scheme 7:**
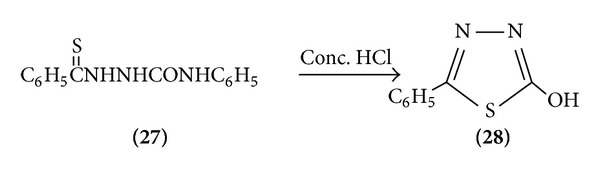


*(b) From Semicarbazide*. When 4-phenyl-1-(thiobenzole) semicarbazide reacts in the presence of concentreted HCl giving 2-hydroxyl-5-phenyl-1,3,4-thiadiazole [13] (Scheme 7).

*(c) From Thiosemicarbazide*. Many syntheses of the 1,3,4-thiadiazole proceed from thiosemicarbazide or substituted thiosemicarbazide.

**Scheme 8:**
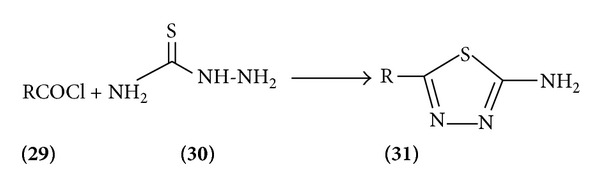


*Method 1*. Gupta et al. have shown that thiosemicarbazide cyclizes directly to 2-amino-5-diazole with acetyl chloride. This simple route to 2-amino 5-substituted-1,3,4-thiadiazole seems to be quite general. In the example shown, R may be methyl17, nor hydnocarpyl, benzyl, cyclopropyl [14], and many others (Scheme 8).

**Scheme 9:**
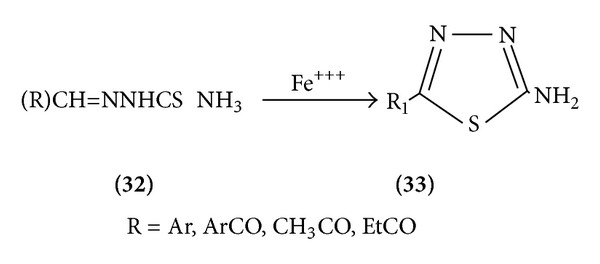


*(d) From Thiosemicarbazone*. 2-Amino-5-substituted thiadiazoles are prepared by oxidative cyclization of thiosemicarbazones with ferric chloride found 1,3,4-thiadiazole [15] (Scheme 9).

**Scheme 10:**
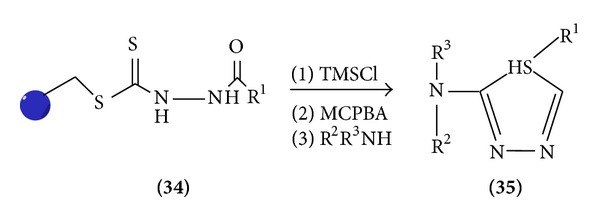


*(e) From Resin*. Resin with TMSCl, MCPBA, and R^2^ R^3^ NH gave 1,3,4-S thiadiazole [16] (Scheme 10).

*(f) From Bithioureas*. Bithiourea and substituted bithiourease have been converted to 1,3,4-thiadiazole by several methods.

**Scheme 11:**
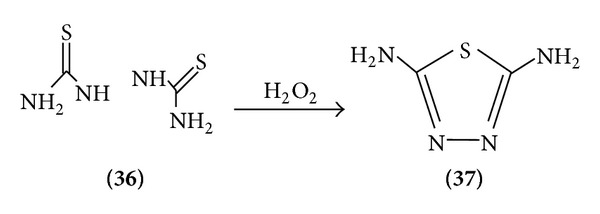


*Method 1*. Bithiourea when treated with 3% hydrogen peroxide is cyclized to 2,5-diamino-1,3,4-thiadiazole (Scheme 11).

*Method 2*. Acetic anhydride acts on bithiourea to form a diacetyl derivative of 2,5-diamino-1,3,4-thiadiazole. The acetyl group I is easily removed by hydrolysis to give the parent thiazole [17]. 

### 5.2. Chemical Properties

#### 5.2.1. Reactivity

Some of the characteristic reactions of the 1,3,4-thiadiazole nucleus are ring opening by strong base ease of nucleophilic attack and the formation of mesoionic compounds by quaternization. The substituents in the 2 and 5 positions have a large effect in determining the reactivity of the molecule as a whole. Thus, the ambient nucleophilicity of 2-aminothiazoles gives rise to electrophilic attack on both the amino group and the nuclear nitrogen atom. Ring formation between these two nitrogen atoms is also a common reaction. 2-Mercaptothiazoles react similarly to arenethiols while a methyl group on the thiadiazole ring has reactivity similar to that in a picoline. Nucleophiles easily displace halogen atom from the thiadiazole nucleus. This is due to the electronegativity of the two nuclear nitrogen atoms which impart a low electron density to the carbon atom of the nucleus.

#### 5.2.2. Thermal and Photochemical Reactions

**Scheme 12:**
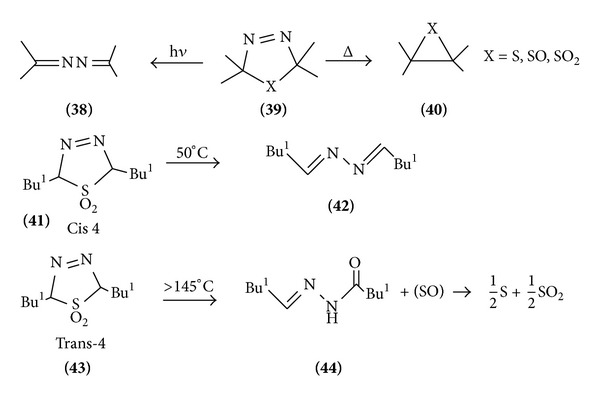


Thermal and photochemical fragmentations of 1,3,4-thiadiazole often follow the fragmentation pattern observed in the mass spectrometer. The cis-2,5-di-t-butyl-2,5-dihydro-1,3,4-thiadiazole gives while photolysis yields the katazine. The cis 2,5-dihydro-1,3,4-thiadiazole 1,1-dioxide (cis-4) undergoes thermolysis at 50°C to give the azine. The trans isomer (trans-4), however, undergoes thermolysis only above 145°C to give the alkylidine hydrazide plus sulphur monoxide which disproportionate into sulphur dioxide. Compounds were independently prepared from dimethylpropanyl, and the hydrazole thermolysis of the isomeric 2,3-dihydro-1,3,4-thiazole 1,1-thiadiazole 1,1 dioxide also gives rise. The concerted [4+1] cycloelimination of sulphur dioxidefrom the trans isomer cannot take place due to steric hindrance (Scheme 12).

*(1) Reaction with Electrophile*. Because unsaturation rings have excessive *π*e^−^, the two nitrogen atoms e^−^ are pulled towards them and ring carbon atoms remain with low e^−^ density and consequently no electrophilic substitution possible in unsubstituted 1,3,4-thiadiazole ring. 

**Scheme 13:**
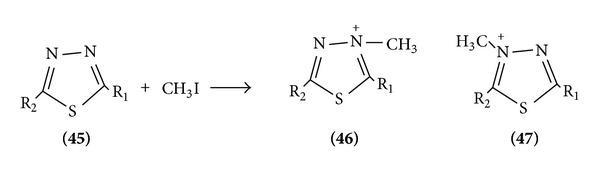


*(a) Attack at Ring Nitrogen (Quaternization)*. Ratio of 3 or 4 substituted product depends on ring substitution, in which nitrogen has high e^−^ density (Scheme 13).

**Scheme 14:**
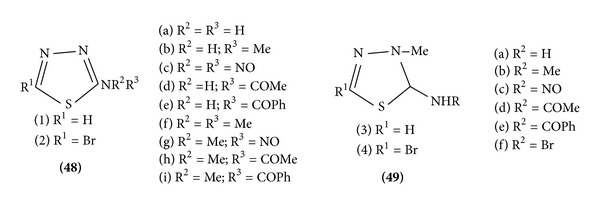


*(b) Electrophilic Attack at Carbon*. Due to the electron density at the carbon atoms in 1,3,4-thiadiazole, such reaction as nitration, acetylation, sulphonation, halogenations. However, 2-amino-substituted 1,3,4-thiadiazoles (1a–i) heat with bromine in acetic acid to give 5-bromo derivatives (2a–i). Similarly, the thiadiazoles (3b) yield (3d–f) the corresponding 5-bromo derivatives (4b) and (4d–f) (Scheme 14).

**Scheme 15:**
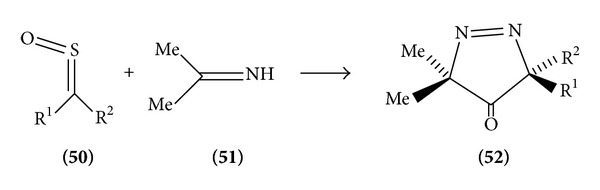


*(c) Electrophilic Attack at Sulphur*. Although direct oxidation at sulphur atom in 1,3,4-thiadiazoles has not been reported, Δ^3^ 1,3,4-thiadiazole 1-oxides and 1,1 dioxides can be indirect mean (Scheme 15).

*(d) Electrophilic Substitution at Ring*. Strong e^−^ donar substituted at ring position 2 activates towards electrophilic agents under drastic condition (conc. H_2_SO_4_ + fuming HNO_3_).

**Scheme 16:**
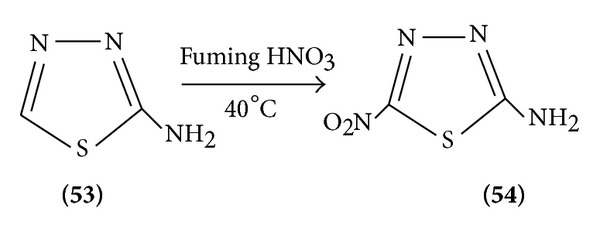


Nitration of 2 ammonia 1,3,4-thiadiazoles with fuming nitric acid at 40°C gave 2-amino-1,3,4-thiadiazoles (21A) (Scheme 16).

### 5.3. Amination

**Scheme 17:**
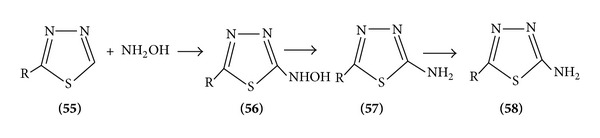


**Scheme 18:**
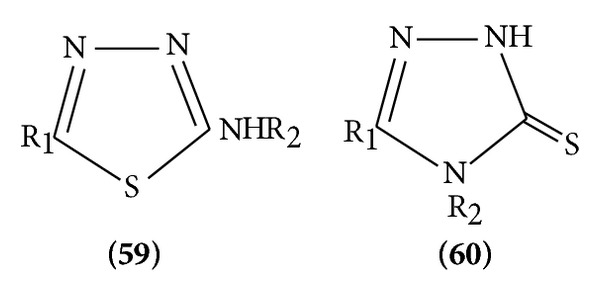


Direct nuclear amination of certain thiadiazoles is possible, illustrating the case of nucleophilic attack. (R=H) react with hydroxylamine in the presence of base to give (R=NH_2_) presumably via the imine (Scheme 17).

(III) *Nucleophilic Attack*. 1,3,4-Thiadiazole ring reacts with strong nucleophiles, treatment of the parent compound with base leading to ring fission. 2-Amino and 2-methylamino 1, 3, 4-thiadiazole rearrange in the presence of methylamine to the triazolinethione (Scheme 18).

**Scheme 19:**
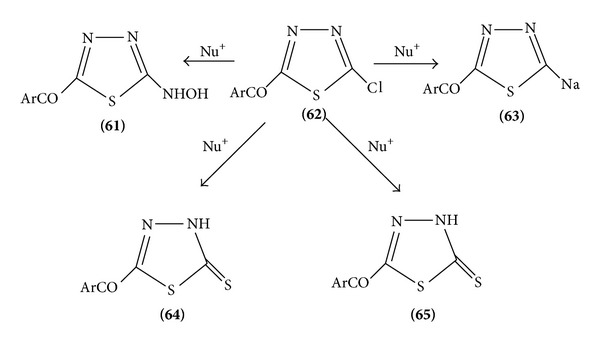


(2) *Nucleophilic Substitution*. Due to the electronegativity of the two nitrogen atoms in the ring, the carbon atom has low electron density and gives many possible nucleophilic reactions [18]. Halogenate 1,3,4-thiadiazoles are important intermediates in which the halogen atom is readily by nucleophiles. 5-Chloro-thiadiazole reacts readily with nucleophiles to give a series of 5 substituted-2-aryl-1,3,4-thiadiazoles (Scheme 19).

**Scheme 20:**
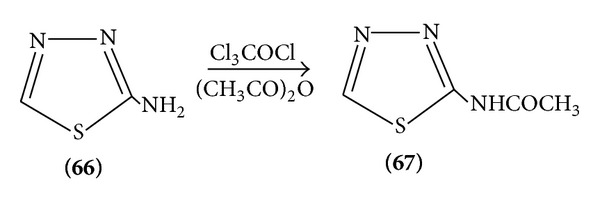


Amino thiadiazoles are rather weak bases; they are nucleophilic enough to be readily acetylated by acid chlorides or acetic acids (Scheme 20).

**Scheme 21:**
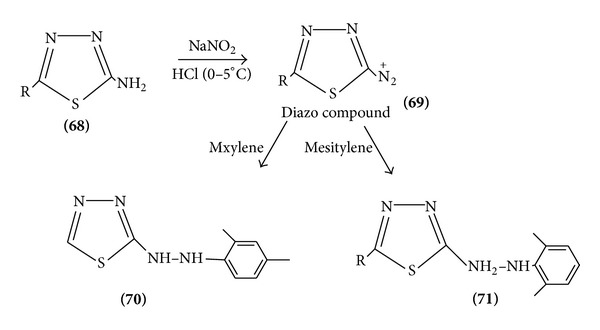


Aminothiadiazole forms diazonium salts and shows the coupling activity [19] (Scheme 21).

**Scheme 22:**
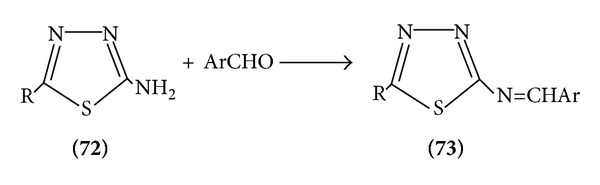


2-Amino-1,3,4-thiadiazoles and aromatic aldehydes form shiff bases [20] (Scheme 22).

### 5.4. Miscellaneous Reaction

**Scheme 23:**
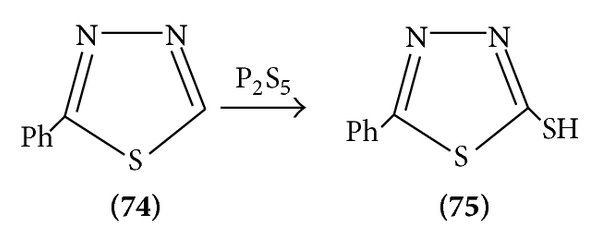


Direct introduction of mercapto group on 2-phenyl-1, 3, 4-thiadiazoles is possible by the use of phosphorous pentasulphide [21] (Scheme 23).

#### 5.4.1. Reduction of Thiadiazole Ring

**Scheme 24:**
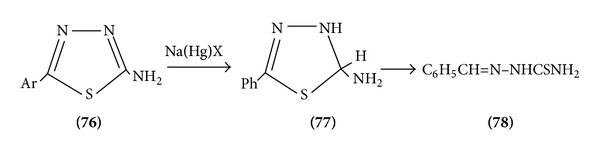


2-Amino-1,3,4-thiadiazoles reduces to benzaldehyde thio semicarbazone [22] (Scheme 24).

#### 5.4.2. Reaction Involving Formation

**Scheme 25:**
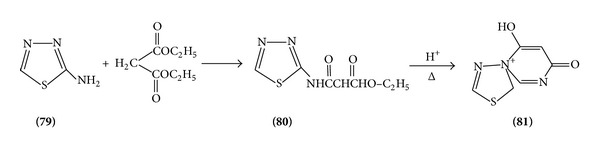


On heating, 2-amino thiadiazole reacts with many dicarbonyl group containing compounds and forms various types of rings attached to thiadiazole (Scheme 25).

#### 5.4.3. Rearrangement Reaction

**Scheme 26:**
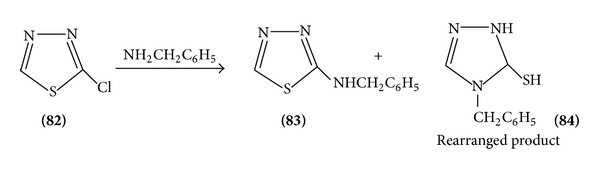


Various 2-chloro and 2-amino substituted thiadiazoles undergo rearrangement by ring opening intermediate formation and again ring closure to form 4N substituted 1,3,4-thiadiazole 5(1)-terione [23, 24] (Scheme 26).

**Scheme 27:**
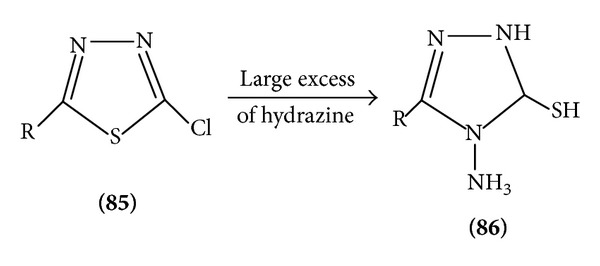


*(a) Dimorph Rearrangement*. See Scheme 27.

**Scheme 28:**
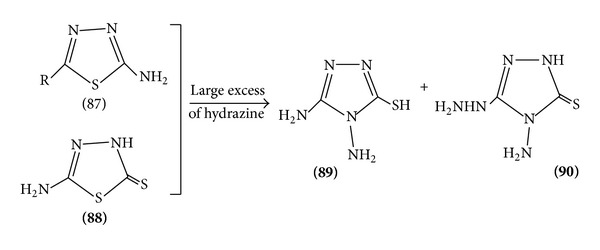


*(b) Under Same Condition*. See Scheme 28.

**Scheme 29:**
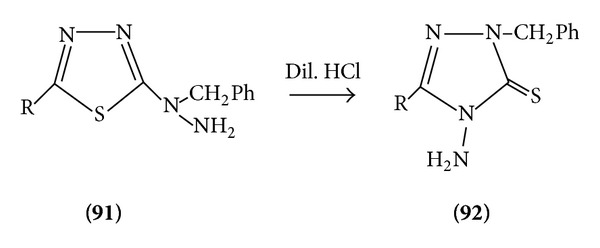


*(c) Acid Catalysed*. See Scheme 29.

**Scheme 30:**
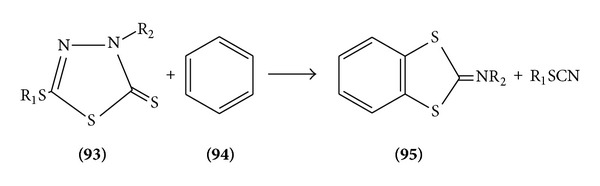


*(d) Cycloaddition Reaction*. Aminobenzothiadiazoles are produced when 1,3,4-thiadiazoleinethiones (R^1^=H, Me, Ac, R^2^=Me, Ph) are reacted with benzyne, most likely by a 1,3-dipolar addition mechanism followed by elimination of RSCN (Scheme 30).

#### 5.4.4. Reaction of Constituents on Carbon

**Scheme 31:**
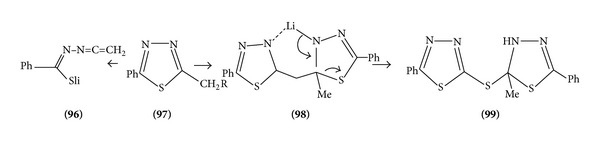


Treatment of 2-methyl 1,5 diphenyl-1-1,3,4-thiadiazole (R=H) with *n*-butyllithium at −78°C gives the lithio derivative (R=Li) which, on treatment with methyl iodide, gives the expected 2-ethyl homologue (R=Me); if (R=Li) is allowed to warm from −78°C to 25°C the dimer is formed, which on heating above 150°C converts into starting material. The dimerization proceeds via intermediates and not the ketone amine (Scheme 31).

#### 5.4.5. Sulphur Substituents

**Scheme 32:**
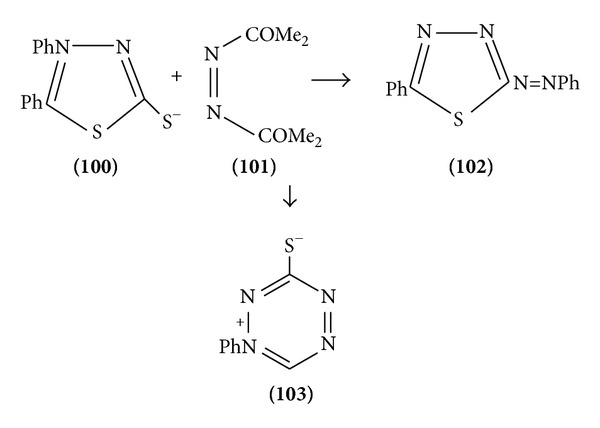


The mesoionic 4,5-diphenyl-1-1,3,4-thiadiazole-2-thiolate reacts with methyl azodicarboxylate to yield the azothiadiazole and not the tetrazine betaine as previously claimed from 2-amino-5-phenyl-1,3,4-thiadiazole and nitrosobenzene (Scheme 32).

#### 5.4.6. Amino Substituents

The reaction of unsubstituted 2-amino-1,3, and 4-thiadiazole with 1,3-dicarbonyl compounds is independent of the nature of the dicarbonyl compounds. The reaction of pentane-2-4-dione gives the 4,6-dimethyl 1,2-thiocyanatepyrimidine. The formation may proceed via the cation. With ethylacetoacetate, however, if a mixture formed also being converted in to on heating. 

*Biological Activities of 1,3,4-Thiadiazole Derivatives*. During the recent years, there has been intense investigation of different classes of thiadiazole compounds, many of which possess extensive pharmacological activities. Among of these compounds having 1,3,4-thiadiazole nucleus are known to exhibit unique antimicrobial activity [25–38]. Antifungal [39–41], antidiabetic [42], anti-inflammatory [38, 43–54], antileishmanial activity [55–58], antituberculosis activity, [59–61], anticancer activity, [34, 62–68] anti-HIV activity [69, 70], antioxidant/radioprotective Activity [71–73], Carbonic anhydrase inhibitors [74, 75]. Anti-*Helicobacter pylori* activity [76, 77]. This paper focuses on the therapeutic importance of novel thiadiazole derivatives as anticonvulsant agents for future. 

*Anticonvulsant Activity of Thiadiazole Derivatives*. A series of 3-aryl amino/amino-4-aryl-5-imino-D2-1,2,4-thiadiazole has been synthesized and screened for anticonvulsant activity. All the synthesized compounds were evaluated against maximal electroshock-induced seizure (MES) and subcutaneous-pentylenetetrazole- (ScPTZ-) induced seizure models in mice. Among the compounds tested, all showed protection from MES seizures, whereas only **(104) (105)** was found to be active in the ScPTZ test. The present results revealed that a number of 3-aryl amino/amino-4-aryl-5-imino-D2-1,2,4-thiadiazoles exhibit a range of activities in anticonvulsant screen [78].


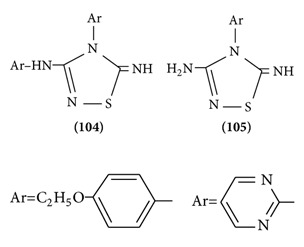


A series of new substituted 1,2,4-thiadiazoles were synthesized by appropriate route and screened for anticonvulsant, neurotoxic, and sedative-hypnotic activities. The structures of the synthesized compounds were confirmed by IR spectroscopy, C-13 NMR, and elemental (nitrogen and sulphur) analysis. After i.p. injection of the compounds to mice or rate at doses of 30, 100, and 300 mg/kg, body weights were examined in the maximal electroshock-induced seizure (MES) and subcutaneous-pentylenetetrazole- (scPTZ-) induced seizure models after 0.5 and 4 h. All the compounds showed protection against MES screen after 0.5 h. Compounds were active at the doses of 100 mg/kg and 300 mg/kg dose i.p. It may be concluded that the synthesized compounds were potent against MES-induced seizures than ScPTZ-induced seizures [79] (Structure **(106)**).


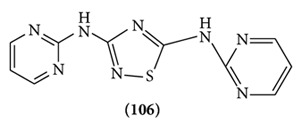


A series of five-membered heterocyclics were synthesized by the reaction between isoniazid and various substituted isothiocyanates and tested for their anticonvulsant activity by determining their ability to provide protection against convulsions induced by electroconvulsiometer. Among the synthesized compounds, (**107f**) 2-(4-chlorophenyl) amino-5-(4-pyridyl)-1,3,4-thiadiazole and (**108f**) 2-(4-chlorophenyl)amino-5-(4-pyridyl)-1,3,4-oxadiazole were found promising compounds of the series [80].


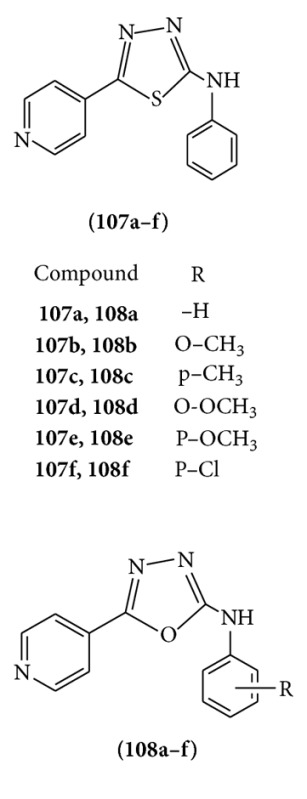


A series of aromatic aldehyde imine derivative of 2-thiobezyl-1,3,4-thiadiazole were synthesized. These derivatives **109(a–e), 110(ia–ie), 110(iia–iie) **show good anticonvulsant activity. Among these compounds, chlorobenzyl substituted compound shows the potent anticonvulsant activity against MES method [81].


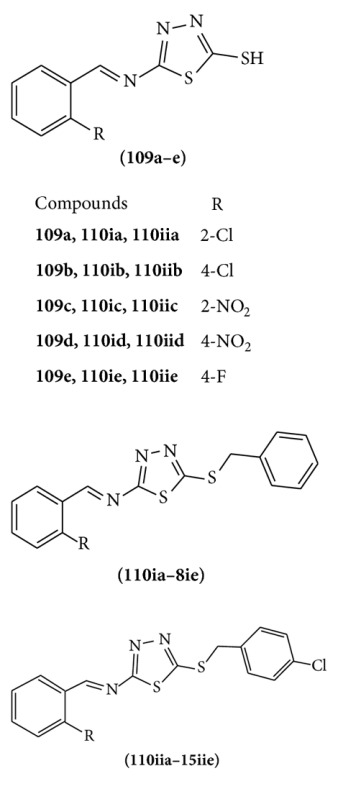


A variety of new 3-[5-substituted phenyl-1,3,4-thiadiazol-2-yl]-2-styryl quinazoline-4(3*H*)-ones were synthesized and evaluated for anticonvulsant activity b. Compounds were examined in the maximal-electroshock- (MES-) induced seizures and subcutaneous-pentylenetetrazole- (scPTZ-) induced seizure models. Compounds **(111a)**, **(111b)**, and **(111c)** showed good anticonvulsant activity in the test models [82].


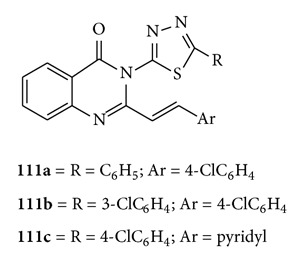


Two new series of 2,5-disubstituted-1,3,4-thiadiazoles were synthesized for their possible anticonvulsant, antibacterial, and antifungal activities. The degree of protection afforded by these compounds at a dose of 100 mg/kg i.p. against pentylenetetrazole-induced convulsions in mice ranged from 0% to 90%. Among these compounds, **112a** (90%) and **112g** (70%) showed maximum protection [83].


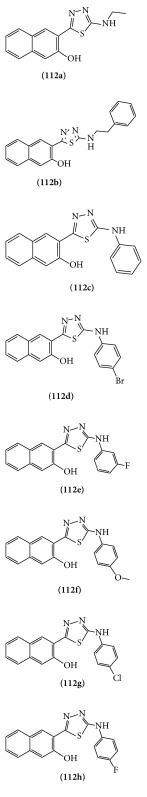


3-[(2-Methyl-1*H*-3-indolyl)methyl]-4-aryl-4, 5-dihydro-1*H*-1,2,4-triazole-5-thiones and their respective *N*-{5-[(2-methyl-1*H*-3-indolyl)methyl]-1,3,4-thiadiazol-2-yl}-*N*-arylamines have been prepared. Behavioral effects, induced by the members of both series, in conjunction with their activity in some specific tests (forced swim, pentetrazol convulsions) on mice, show that these derivatives cross the blood-brain barrier and could develop an antidepressant activity comparable to that of imipramine. Blood-brain barrier penetration is also supported by the lipophilicity data obtained for all analogs [84]. (Structure **(113)**).


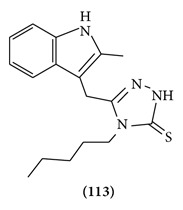


Several novel 2-amino-5-[4-chloro-2-(2-chlorophenoxy)phenyl]-1,3,4-thiadiazole derivatives 4a–d were synthesized, and their anticonvulsant activity was determined by evaluation of the ability of these compounds to protect mice against convulsion induced by lethal doses of pentylenetetrazole (PTZ) and maximal electroshock (MES). The result of anticonvulsant data shows that among the synthesized compounds, 5-[4-chloro-2-(2-chlorophenoxy)phenyl]-*N*-ethyl-1,3,4-thiadiazol-2-amine **(114**, **115**, **116)** was the most active compound in both MES and PTZ tests with an ED50 of 20.11 and 35.33 mg/kg, respectively [85].


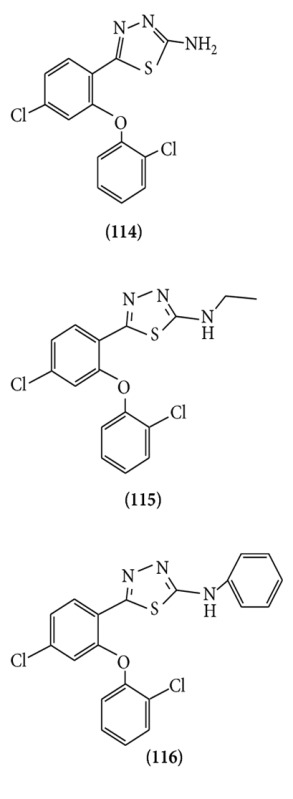


The present study describes the synthesis and anticonvulsant activity evaluation of 6-substituted-[1,2,4]triazolo[3,4-b][1,3,4]thiadiazole derivatives and their partially dehydrogenated products 5,6-dihydro-6-substituted-[1,2,4]triazolo[3,4-b][1,3,4]thiadiazole derivatives. The bioevaluation demonstrated that most compounds in the series exhibited potent anticonvulsant activity in the maximal electroshock test. Among which, 6-(4-chlorophenyl)-[1,2,4]triazolo[3,4-b][1,3,4]thiadiazole **(117)** emerged as the most promising candidate on the basis of its favorable ED50 value of 23.7 mg/kg and PI value of 10.8. In addition, the potency of compound **117** against seizures induced by pentylenetetrazole, 3-mercaptopropionic acid, and bicuculline in the chemical-induced seizure tests suggested that compound **117** displayed broad-spectrum activity in several models, and it may exert its anticonvulsant activity through affecting the GABAergic system [86].


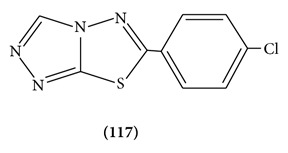


 Synthesis and pharmacological evaluation of a number of substituted 1, 3,4-thiadiazole the first member of the series 2-(aminomethyl)-5-(2-biphenyl)-1,3,4-thiadiazole **(118)** was found to possess potent anticonvulsant property in rats and mice and compared favourable with the standard anticonvulsant drug phenytoin, Phenobarbital and carbamazepine in a number of test situations. The potency of compound was maintained on alkylation of the side chain nitrogen atom; however, aryl substitution on chain lengthening caused a drop in potency replacement of the two biphenyl group by phenyl or benzyl also lead to inactive compound [87]. 


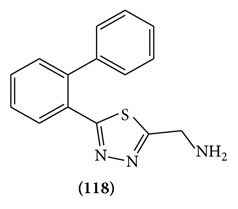


Various N-(5-chloro-6-substituted-benzothiazol-2-yl)-N′-(substituted phenyl)-[1,3,4]thiadiazole-2,5-diamines were designed and synthesized starting from substituted acetophenones. Structures of all the compounds were confirmed on the basis of spectral and elemental analyses. All the newly synthesized compounds were screened for their anticonvulsant activity and were compared with the standard drug phenytoin sodium. Interestingly, all the compounds showed protections against seizures in the range 50%–100% indicative of the promising nature of the compounds against seizure spread. Compound **119** showed complete protection against MES-induced seizures [88].


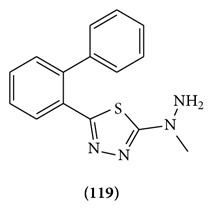


A series of 1,2,4-thiadiazoles **(120a–e)** were prepared and evaluated for anticonvulsant activity by Siddiqui et al. The compound with para-chloro substitution (**120c**) showed maximal activity in MES test and blocked strychnine seizures to some extent whereas other compounds of the series were less active [89].


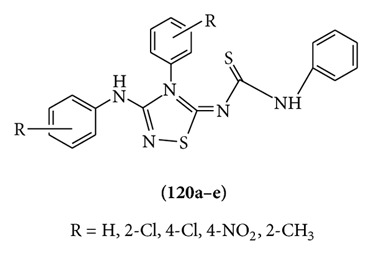


A series of 1-(substituted phenyl)-3-[(5-substituted phenyl)-1,3,4-thiadiazol-2-yl]-2-thioxodihydropyrimidine-4,6 (1*H*,5*H*)-diones **121(a–w)** were designed, synthesized in good yields. The compounds were evaluated for anticonvulsant activity. The compounds were potent in MES test and were less neurotoxic as compared to standard drug phenytoin [90].


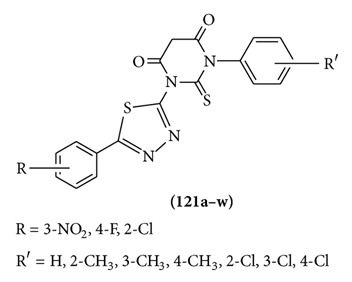


A series of thiadiazole derivatives were synthesized with differently substituted benzoic acids which were cyclized to give differently substituted thiazolidin-4-one. Elemental analysis, IR, HNMR,C NMR, and mass spectral data confirmed the structure of the synthesized compounds. The derivatives of these moieties were evaluated for anticonvulsant activity by MES model and neurotoxicity by rotarod method. The synthesized compounds showed good potential for anticonvulsant activity besides this, and the compounds also showed neurotoxic effect. It was observed that compounds with OCH3 at 3, 4 position of phenyl ring showed less protection against convulsions as compared to compounds having unsubstituted phenyl ring [91].

The synthesis and anticonvulsant activity of a series of 2-aryl-5-hydrazino-1,3,4-thiadiazoles are described. The combination of preferred aromatic substituents in the 2-position coupled with alkyl substitution on the hydrazine moiety led to a number of potent compounds lacking sedation, ataxia, or lethality. 5-(2-Biphenylyl)-2-(1-methylhydrazino)-1,3,4-thiadiazole (4m) represents a new class of anticonvulsant agent and compares favorably with the standard drugs phenytoin, phenobarbital, and carbamazepine [92] (Structure **(122)**).


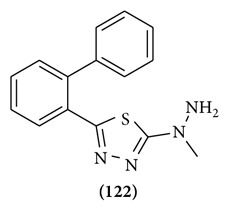


A novel series of NN′-{5-[(1H-indol-3-ylmethyl)-1,3,4-thiadiazol]-2-yl}-N4-(4-substituted benzaldehyde) semicarbazones, N1-{5-[(1H-indol-3-ylmethyl)-1,3,4-thiadiazol-2-yl}-N4-[1-(4-substituted phenyl)ethanone]-semicarbazones and N1-{5-[(1H-indol-3-ylmethyl)-1,3,4-thiadiazol-2-yl}-N4-[1-(4-substituted phenyl) (phenyl) methanone]-semicarbazones were synthesized and evaluated for their anticonvulsant potential using maximal electroshock seizure (MES) and subcutaneous pentylenetrtrazole (scPFZ) models. The minimal motor impairment (neurotoxicity) was determined by rotarod test. The results of the present study confirmed the requirements of various structural features of four binding site pharmacophore model for anticonvulsant activity [93].

Treatment of 2-bromoacetylbenzofuran with 1*H*-benzotriazole afforded 1-(benzofuran-2-yl)-2-(benzotriazol-1-yl) ethanone which reacted with phenylisothiocyanate to give the corresponding thioacetanilide derivatives. Treatment of the latter ethanone and thioacetanilide derivatives with hydrazonoyl chlorides afforded the corresponding pyrazole and 1,3,4-thiadiazolederivatives. The thioacetanilide derivative reacted with *α*-haloketones and *α*-halodiketones to afford thiophene and thiazole derivatives, respectively. The newly synthesized compounds were found to possess anticonvulsant and anti-inflammatory activities with the same mechanism of action of selective COX-2 inhibitors [94].

## Figures and Tables

**Table 1:** Physical properties of 1, 3, 4-thiadiazole.

Bond length		Bond angle	
Type	Å	Type	(°)
a	1.371	*α*	112.2
b	1.302	*β*	114.6
c	1.721	*γ*	86.4
d	1.721	*δ*	114.6
e	1.302	*ε*	112.2
